# Metropolitan and Provincial Schools of Medicine

**Published:** 1905-09-02

**Authors:** 


### Metropolitan and Provincial Schools of Medicine.

As the first of October draws near, the question whether a young man intended for the medical profession is to obtain his education at a metropolitan or at a provincial school is one which will be anxiously considered by many parents or guardians, and the decision of which, in many circumstances, may exercise an abiding influence upon the student's career. As we have written elsewhere, education for the medical profession may be roughly divided into the two branches of the preliminary and the clinical, and there can be no doubt that for the former of these some provincial schools have of late years possessed advantages over some metropolitan ones. The staffs of all the hospitals of a great provincial city have combined for the purpose of affording the best attainable preliminary instruction, while the staff of every large London hospital has, as a rule, sought to supply whatever was required from its own body. In this respect the altered relations between the University of London and some of the hospitals, to which we also refer elsewhere, will be likely soon to produce changes altogether in favour of the Metropolis, which is always likely to have the largest aggregate of students and, therefore, the greatest power of offering a tempting position to professors. In other respects the advantages claimed for provincial schools have usually been a lesser degree of separation from home, with correspondingly greater facilities for the supervision of life and conduct, smaller cost both of living and of instruction, and the prospect of strengthening or of forming ties which might lead afterwards to connection with the local hospitals, and through them to provincial reputation and success. None of these considerations are to be ignored; although, with regard both to supervision and to expense, we should be much inclined to think that the advantages to be expected have been in some degree exaggerated. In the first place, when a young man is destined for such a profession as the medical, in which he will constantly be called upon to exercise independent judgment in conditions involving great responsibility, the sooner he is left to feel his own feet the better. If he cannot be trusted to guide himself as a student, he is very little likely ever to be fit to guide others as a practitioner. With regard to expense, we doubt the wisdom of bringing into the profession any young man who does not possess sufficient capital to secure to him a fair start in practice; among the conditions of which fair start we should assign a prominent place to a thoroughly good education, alike preliminary and professional, neither of them to be attained without expenditure. The advantages of the provincial school as a source of local connection may often be unquestionable, but perhaps only when there is either considerable family influence or a high degree of talent. The perception of only middling talent, on the part of friends and neighbours, is apt not to be sufficiently decided to exert great influence upon a career. When other considerations are laid aside there remains the great fact of the enormous wealth of the metropolis in clinical material and in professional and tutorial ability. Liverpool is the only place which can compete with London in its supply of patients suffering from those tropical diseases which are daily assuming greater and greater importance, whether from an individual or from an imperial point of view; and, with regard to all others, London stands alone. It stands alone, also, in the circumstance that the number of its eminent men is too great to allow of the pre-eminence of any; and that the students trained in its schools are therefore more apt than others to think for themselves, and are comparatively free from the temptation *jurare in verba magistri.*

